# Identification of candidate serum protein biomarkers in Down's syndrome dementia

**DOI:** 10.1002/alz70856_102904

**Published:** 2025-12-25

**Authors:** Ruma Raha‐Chowdhury

**Affiliations:** ^1^ University of Cambridge, Cambridge, United Kingdom

## Abstract

**Background:**

Down syndrome (DS) individuals develop amyloid plaques and an increased risk of developing Alzheimer's dementia (DSAD). The detection of pre‐symptomatic DSAD when potential disease‐modifying therapeutic interventions are most likely to yield benefit can be achieved by developing biological markers that can serve as supportive early screening for the detection of AD before it is clinically evident.

**Method:**

Blood samples from well characterized DS subjects and age matched controls (*n* = 50, age 39 ± 26 years) who have had brain PET scan to detect fibrillar amyloid‐beta using [11C]‐Pittsburgh Compound‐B‐PET scanning were analyzed. A centrifugation method was developed to isolate fibril binding proteins from serum and analyzed by MALDI‐TOF mass spectrometry (MOLDI‐MS). Serum protein levels were measured by Western blotting or ELISA. Significant findings were evaluated in DS brain tissues by in‐situ hybridisation and Immunohistochemistry.

**Result:**

MOLDI‐MS identified eighty‐five proteins represented by multiple tryptic peptides, twenty‐five of which showed significant differences between DS and controls, confirmed by WB and ELISA analysis. Those differentially expressed proteins are amyloid b, afamin, a1‐microglobulin, apolipoprotein C‐II, apolipoprotein E, ABCG1, ceruloplasmin, complement (C1r, C1q), clusterin, cystatin‐C, defensin, DPPIV/CD26, ferritin, hepcidin, histone, tau, themis, TREM2, transthyretin, serum amyloid P, myocardin‐like protein 2, neuronal pentraxin receptor, retinol binding protein‐4 (RBP4) and TGFb. CD26 expression patterns reduced in the DS brain, amyloid b and transthyretin, were significantly up‐regulated, clusterin and retinol binding protein‐4 were down‐regulated in the DS serum. Statical analysis was evaluated by ANOVA (*** =p<0.001and **** =p<0.0001).

**Conclusion:**

Several of the differentially expressed proteins identified in this study have previously been linked as risk factors for Alzheimer disease and are components of functional pathways thought to contribute to the pathogenesis of AD and DSAD. These pathways include immune cell activation, reverse cholesterol transport, regulator of iron homeostasis and inhibition of Ab fibrils and oligomers. Furthermore, the significant difference of concentrations of these proteins in the sera of DS individuals may be further developed to generate non‐invasive methods to identify and track the progression of AD, even in the prodromal phases.

